# Application of Operational Tolerance Signatures Are Limited by Variability and Type of Immunosuppression in Renal Transplant Recipients: A Cross-Sectional Study

**DOI:** 10.1097/TXD.0000000000000638

**Published:** 2016-12-21

**Authors:** Matthew J. Bottomley, Mian Chen, Sue Fuggle, Paul N. Harden, Kathryn J. Wood

**Affiliations:** ^1^ Oxford Kidney Unit, Churchill Hospital, Oxford, United Kingdom.; ^2^ Nuffield Department of Surgical Sciences, University of Oxford, Oxford, United Kingdom.; ^3^ Transplant Immunology and Immunogenetics, Oxford Transplant Centre, Churchill Hospital, Oxford, United Kingdom.

## Abstract

**Background:**

Renal transplant recipients (RTR) frequently develop complications relating to chronic immunosuppression. Identifying RTR who could safely reduce immunosuppression is therefore highly desirable. We hypothesized that “signatures” described in RTR who have stopped immunosuppression but maintained stable graft function (“operational tolerance”) may enable identification of immunosuppressed RTR who are candidates for immunosuppression minimization. However, the effect of immunosuppression itself on these signatures and circulating B-cell populations is currently unknown.

**Methods:**

We undertook a cross-sectional study of 117 RTR to assess the effect of immunosuppression upon circulating B cell populations, humoral alloresponse and 2 previously published “signatures” of operational tolerance.

**Results:**

Immunosuppression associated with alterations in both published “signatures.” Azathioprine associated with a decrease in transitional and naive B-cell numbers and calcineurin inhibition associated with an increase in the number of circulating plasmablasts. However, only azathioprine use associated with the presence of donor-specific anti-HLA IgG antibodies. Calcineurin inhibition associated with an increase in total serum IgM but not IgG. Data were corrected for age, time since last transplant, and other immunosuppression.

**Conclusions:**

Current signatures of operational tolerance may be significantly affected by immunosuppressive regimen, which may hinder use in their current form in clinical practice. Calcineurin inhibition may prevent the development of long-lasting humoral alloresponses, whereas azathioprine therapy may be associated with donor specific antibody development.

## Abstract 2

Supplemental digital content is available in the text.

Renal transplantation is the gold standard treatment for end-stage renal failure. However, improvements in short-term outcomes have not clearly translated to greater long-term transplant survival.^1-4^ Chronic immunosuppression is a major obstacle to long-term allograft survival due to nephrotoxicity and increased risk of malignancy, infection, and cardiovascular disease.^5^

Immunosuppression minimization could reduce the burden of posttransplant morbidity but in most renal transplant recipients (RTR) may risk an alloreactive immune response potentially leading to alloantibody production and graft rejection. A small cohort of RTR worldwide have stopped immunosuppression and maintained stable prolonged graft function.^6,7^ This is termed “spontaneous operational tolerance” (SOpT).^8^ Prospective identification of immunosuppressed RTR with SOpT may facilitate safe and directed immunosuppression minimization.

Two collaborations (“Reprogramming the Immune System for the Establishment of Tolerance” [RISET] and “Immune Tolerance Network” [ITN]) have independently reported a number of phenotypic changes in circulating blood, termed ‘signatures’, in RTR displaying SOpT.^9,10^ The RISET signature consisted of a cross-platform signature using lymphocyte subsets, whole blood gene expression (quantitative polymerase chain reaction) and direct pathway alloresponsiveness.^10^ The ITN signature used the expression of 3 genes.^9^ These signatures were cross-validated and found to have a sensitivity and specificity of over 80% for the identification of RTR displaying SOpT. The authors proposed that these signatures could potentially identify RTR taking maintenance immunosuppression who may exhibit subclinical SOpT. These signatures and subsequent work suggest that SOpT may be associated with alterations in B cell phenotype and function.^11-15^

We and others have recently published data suggesting that azathioprine may impact upon circulating B cell populations by depleting naïve and transitional B cell subsets.^16,17^ These were univariate analyses and so do not account for other immunosuppressive agents or other potential confounders. We resolved to assess this association more robustly through multivariate assessment. For the first time, we assessed the effect of these agents upon the previously reported RISET and ITN “signatures” of SOpT and the generation of donor-specific anti-HLA antibodies (DSA) in a long-term RTR cohort.

## PATIENTS METHODS

Full methods are detailed in the **SDC,** http://links.lww.com/TXD/A32. The conduct of the study was approved by an National Health Service (NHS) research ethical committee before commencement (reference 12/WS/0288) and was conducted according to the principles of the Declaration of Helsinki. Written consent was provided before enrolment. The study is reported according to STROBE guidelines.

### Patient Recruitment and Clinical Data Collection

Stable long-term RTRs without recent noncutaneous malignancy were recruited at routine transplant outpatient clinic follow-up during the period March 2013 to November 2014. Clinical data were collected using medical and transplant records and pathology results. Estimated glomerular filtration rate (eGFR) was calculated using the 4-variable “modified diet in renal disease” equation.^18^ Information relating to HLA type was not recorded locally for 4 donor-recipient pairs and 5 donors: this information was kindly provided by the NHS Blood and Transplant service.

### Peripheral Blood Mononuclear Cell Extraction and Lymphocyte Phenotyping

Peripheral blood mononuclear cell were extracted from chilled blood within 4 hours of venepuncture. Peripheral blood mononuclear cells were isolated by density-gradient centrifugation and stained using a cocktail of antibodies (**Table S2, SDC,** http://links.lww.com/TXD/A32). Data were acquired using a Navios flow cytometer and analyzed using Kaluza version 1.4 (both Beckman Coulter, Wycombe, UK) and FlowJoX (TreeStar, Inc).

### RNA Isolation and Gene Expression Analysis

Total RNA was extracted from thawed whole blood stored in RNA stabilisation solution (‘Tempus’ tubes, Life Technologies, Paisley, UK) at −80°C using a magnetic bead (“MagMAX”; Life Technologies) method according to manufacturer’s instructions. RNA was stored at −80°C before reverse transcription.

Complementary DNA (cDNA) was generated using a starting quantity of 1 μg of total RNA. quantitative/real-time polymerase chain reaction was undertaken on 30 ng cDNA in duplicate using either inventoried assays or using custom primers and probes (**Table S3, SDC,** http://links.lww.com/TXD/A32). Relative gene expression was normalized to β-glucuronidase using the 2^−ΔCq^ method. No template controls were run in parallel.

### Anti-HLA Antibodies Detection and Definition

Anti-HLA antibodies were detected using solid-phase Luminex bead assays (One Lambda Inc., Canoga, CA), according to the validated protocols used for clinical samples by the Transplant Immunology and Immunogenetics Laboratory, Churchill Hospital, Oxford. Samples were analyzed using a Luminex 100 IS fluorescence detector system (Luminex Corp., Austin, TX).

All samples were first assessed using LabScreen Mixed Screen (LSM12) beads. Samples with a positive result were confirmed using Class I (LS1PRA) or Class II PRA (LS2PRA) beads. If HLA specificities cannot be resolved definitively, further testing was performed using Class I (LS1A04) or Class II (LS2A01) single antigen beads (SAB). Samples were run with positive and negative control samples and control beads. The mean fluorescence intensity (MFI) on each individual sample bead was normalized to a negative control bead within each sample and a negative control sample provided by the manufacturer. For SAB, the cut-off for positivity was an MFI of 1500, representing the clinical threshold used at the Oxford Transplant Centre.

Antibodies to HLA-A, -B, -C, -DR, and -DQ were assessed as this information was available for the majority of participants. For 3 donor-recipient pairs the donor HLA Class II alleles were unknown, so only their HLA Class I data were assessed.

### Serum Immunoglobulin Concentration Quantification

Immunoglobulin concentration in thawed sera was determined by enzyme-linked immunosorbent assay (Total Human IgM and IgG Ready- SET-Go kits, eBioscience) according to manufacturer's instructions.

### Statistical Analysis

Analyses were performed on Graphpad Prism for Windows 5.03 (Graphpad, San Diego, CA) or SPSS 20 (IBM Corp., New York, NY). Continuous variables are reported as median (interquartile range) unless specified otherwise. Hazard ratios are reported as hazard ratio (95% confidence interval). Categorical variables are reported as number (percentage of group).

Intergroup comparison was performed using the nonparametric 2-tailed Mann-Whitney or Kruskal-Wallis tests. For categorical variables the chi-squared test or Fisher exact test were used. Where the Kruskal-Wallis test was significant, a subsequent post hoc Dunn test was applied.

Linear regression for interaction of immunosuppression with the signatures of tolerance was performed using normally transformed variables where appropriate. All variables were transformed using log-transformation. Odds ratios were calculated by logistic regression.

Throughout the study, a *P* value less than 0.05 was considered significant, unless indicated otherwise. To prevent type II (false positive) errors due to multiple testing, where appropriate a Bonferroni correction was applied; the adjusted threshold for significance is indicated where used.

## RESULTS

### Cohort Phenotype

The cohort clinical phenotype is summarised in Table 1. Owing to the long period between transplant and recruitment the majority of participants were receiving cyclosporin, steroid, and/or azathioprine immunosuppression with few participants receiving induction therapy.

**TABLE 1:**
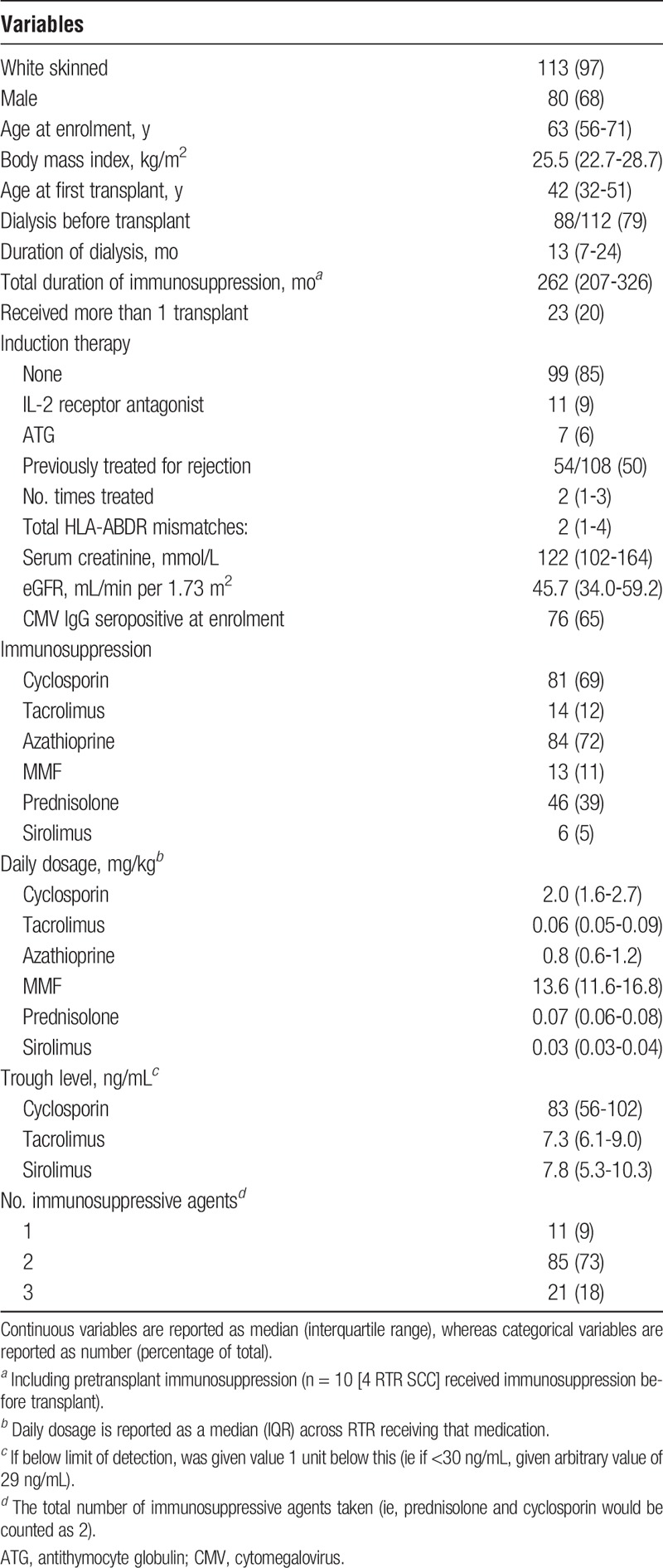
Clinical characteristics of study participants

### Immunosuppression and B-Cell Populations

**FIGURE 1:**
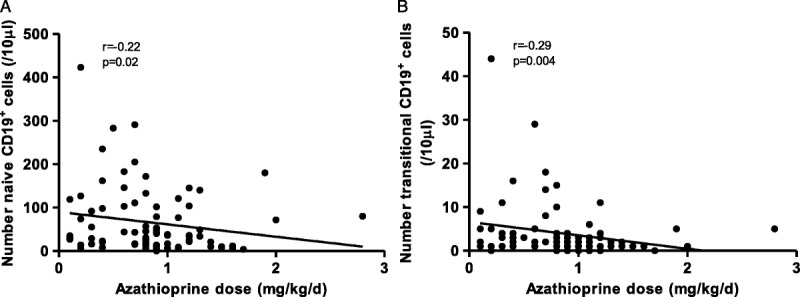
The reduction of circulating naïve and transitional B cell numbers is dose dependent. A) Correlation between naïve B cell number (A), transitional B cell number (B) and daily azathioprine dose (in mg/kg per day). Correlations tested using 2-sided Spearman test, as all 3 variables were nonparametrically distributed. Only RTR taking azathioprine at enrolment were included (n = 82). Both cell populations are given per 10-μL blood.

In support of previous reports, the association between azathioprine and B cell number was dose-dependent. Increasing daily dose of azathioprine correlated inversely with total, naïve and transitional B cell number on univariate analysis (Figure 1).

**FIGURE 2:**
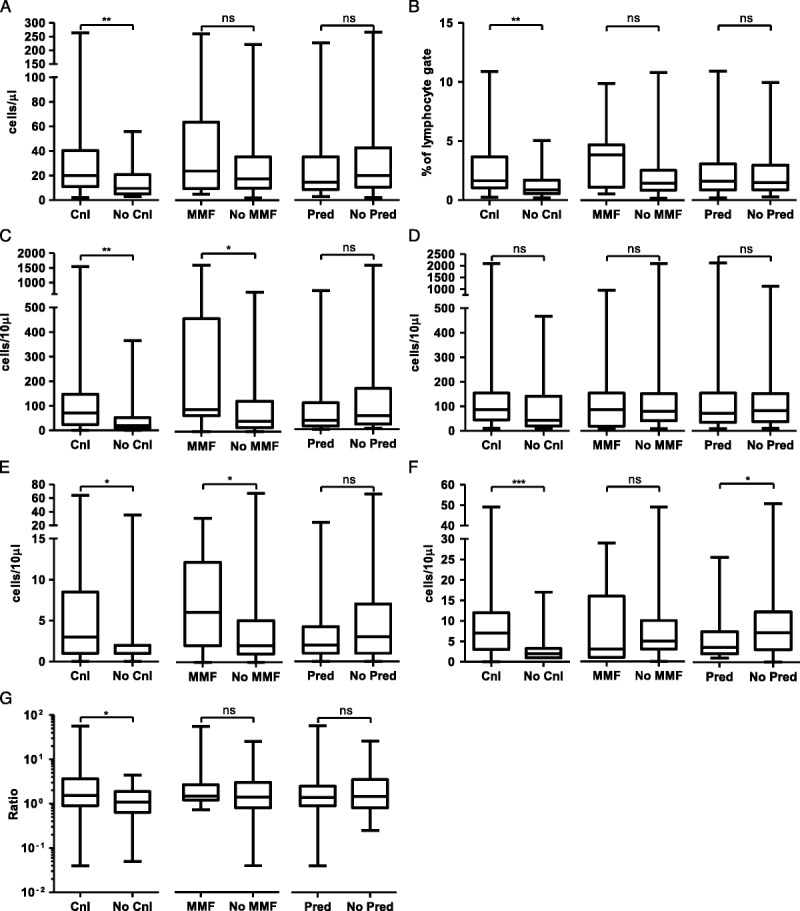
On univariate analysis, calcineurin inhibition, prednisolone and MMF are associated with alterations in circulating B cell populations. A, total CD19^+^ cells; B, proportion of CD19^+^ cells within lymphocyte gate; C, number of CD27^−^IgD^+^ naive CD19^+^ cells; D, number of CD27^+^ memory CD19^+^ cells; En number of CD24^hi^CD38^hi^ transitional CD19^+^ cells; F, number of CD38^hi^IgD^−^ plasmablasts; G, ratio of isotype switched to unswitched CD19^+^ cells. ***P < 0.001, **P < 0.01, *P < 0.05, “ns” not significant by Mann-Whitney test.

On univariate analysis, calcineurin inhibition was associated with an increased number of circulating B cells, with an increase in all B-cell subsets except memory B cells (Figure 2).

To address the issue of polypharmacy linear regression analysis was undertaken to assess the effect of each immunosuppressant individually on the B-cell compartment. Time since last transplant was included to account for changing trends in immunosuppression over time and the potential effect of cumulative immunosuppressive dose. Age at enrolment was also included. Results are shown in Table 2. Azathioprine therapy was associated with decreased naive and transitional B cell numbers, leading to an overall reduction in B cell number. Calcineurin inhibition was associated with alterations within the memory and effector compartments, with increased numbers of plasmablasts and an increased proportion of class-switched memory B cells.

**TABLE 2:**
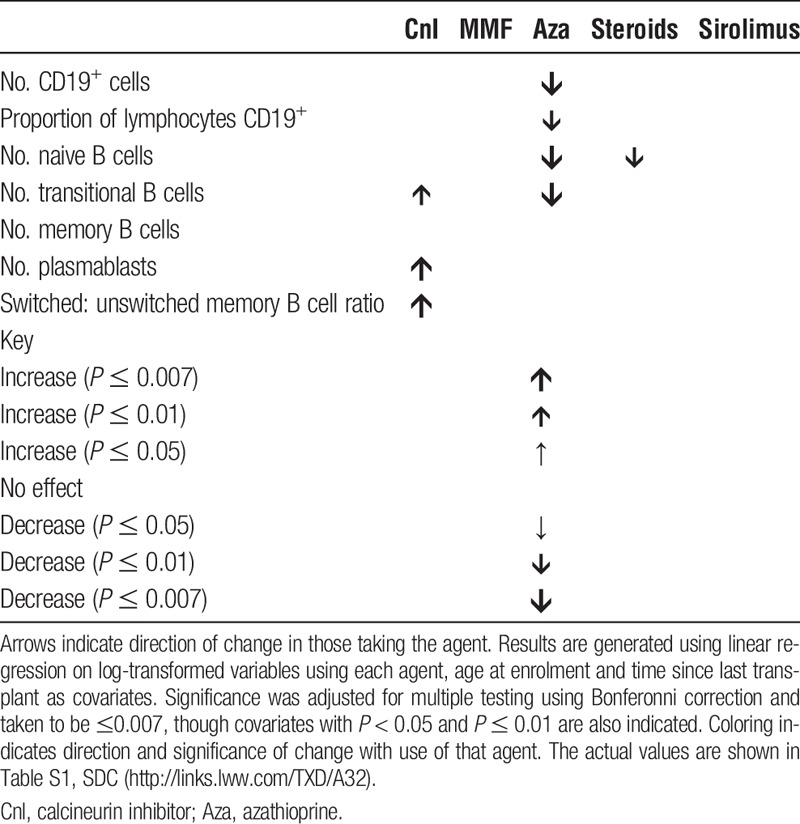
The effect of individual immunosuppressant agents upon the B cell compartment

### Effect of Immunosuppression Upon Indices of SOpT

We next addressed the effect of these alterations in B-cell parameters upon the previously published RISET and ITN signatures.^9,10^

We focused first on the RISET signature. The CD19/CD3 ratio and most genes related to B cells were found to be reduced in RTR taking azathioprine on univariate analysis (Table 3). Those variables which did not relate to B cells, such as the proportion of recently activated T cells, were unaffected. Steroid therapy was associated with a significant elevation in *SLC8A1* and *TLR5* and reduction in *HS3ST1* gene expression on univariate analysis. Conversely, calcineurin inhibition appeared to be associated with a reduction in *SLC8A1* and *TLR5* expression. Mycophenolate mofetil (MMF) use was not associated with any change in these variables (data not shown).

**TABLE 3:**
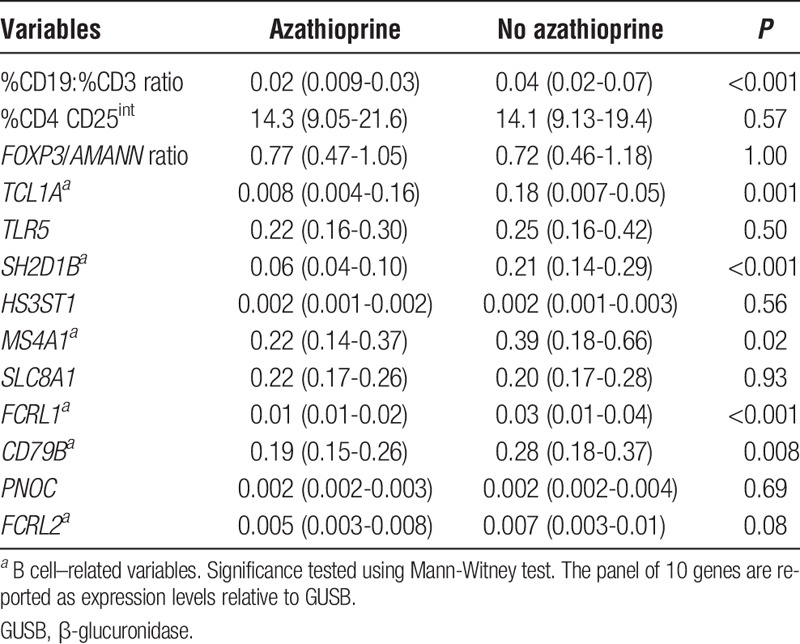
Azathiaprine impacts on the B cell–related variables within the RISET signature of “operational tolerance”

Despite previous data suggesting a reduction in the proportion of regulatory T cell in circulating blood associated with calcineurin inhibition, surprisingly, there was no effect on the *FOXP3*/*AMANN* ratio.^19,20^ This is because a significant reduction in *FOXP3* expression associated with calcineurin inhibition was offset by a significant reduction in *AMANN* expression (**Figure S1, SDC,** http://links.lww.com/TXD/A32).

Linear regression analysis was undertaken as before to assess the impact of immunosuppression upon the RISET signature (Table 4). Upon adjustment azathioprine remained associated with a reduction in CD19:CD3 ratio and a number of B cell-related variables within the gene panel. Corticosteroids were associated with an increase in *TLR5* and *SLC8A1* expression. Only the proportion of recently activated T cells and 1 gene, *FRCL2*, within the gene panel did not demonstrate a link to immunosuppressive regimen.

**TABLE 4:**
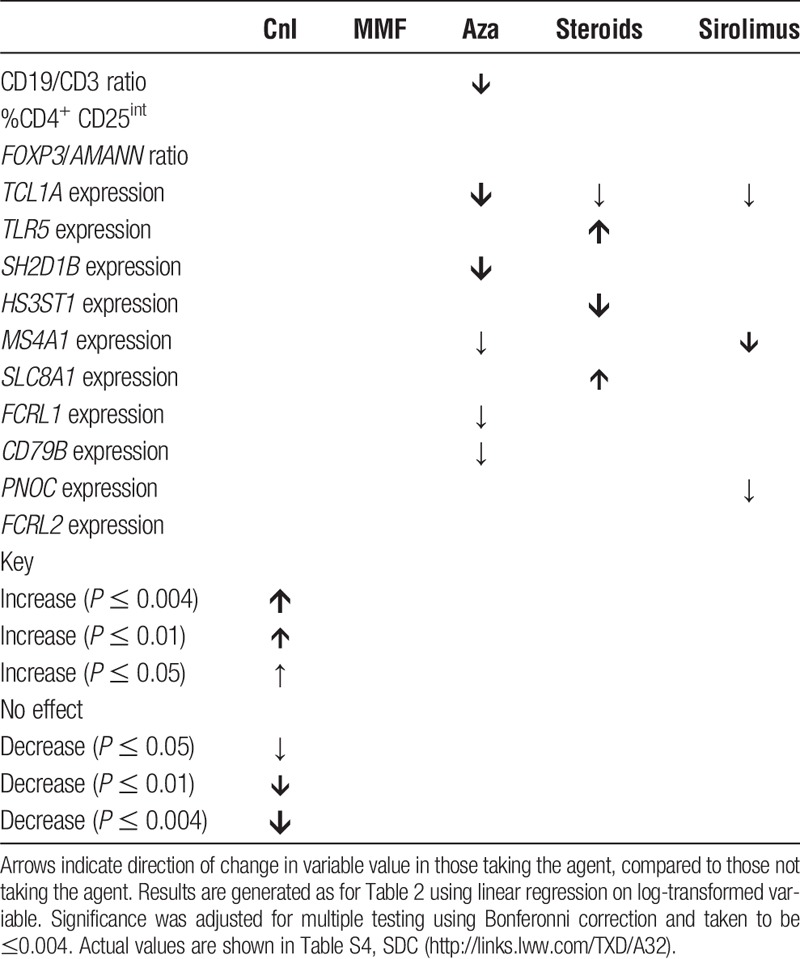
The effect of individual immunosuppression upon the RISET tolerance signature

Given the above, we hypothesized that azathioprine would be associated with a similar effect upon the ITN tolerance signature. As previously reported,^17^ *IGLL1* expression was found to be unreliable and so we focused on the two remaining genes (*IGKV1D-13* and *IGKV4-1*). When the linear regression was repeated, surprisingly calcineurin inhibitor and sirolimus use were linked to alterations in the both variables (Table 5).

**TABLE 5:**
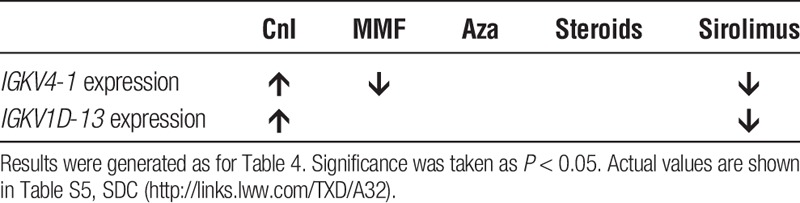
The effect of immunosuppression upon the ITN tolerance signature

### Immunosuppression and Immunoglobulin Production

One of the effector functions of B cells is the production of immunoglobulin. Given the effect of immunosuppression upon circulating B cell populations, we next explored the association between immunosuppression and the presence of donor-specific (anti-HLA) antibodies (DSA). We hypothesized that the increase in plasmablasts and immunoglobulin-related gene expression associated with calcineurin inhibition may also associate with DSA development.

**FIGURE 3:**
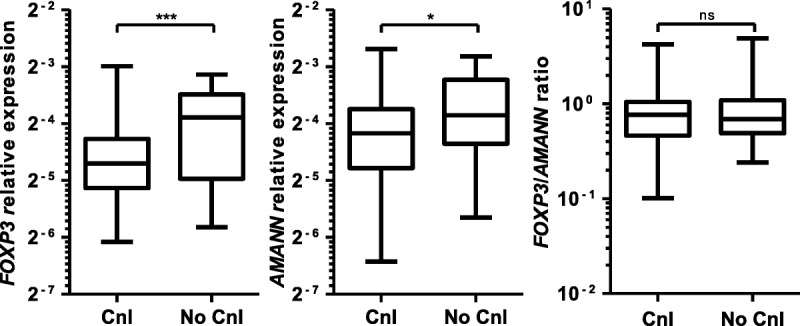
The prevalence and class-specificity of anti-HLA antibodies in the cohort. Numbers of RTR in each category are given.

RTRs were assessed for the presence of anti-HLA Class I and II IgG antibodies using the solid phase Luminex assay (Figure 3).

Twenty-two RTRs, representing 19% of the cohort, possessed DSA; the majority were HLA Class II-specific. 12 (55%) RTR with DSA also demonstrated nDSA, representing a significantly greater proportion than RTR without DSA (n = 27 [28%], p = 0.02). For 17 RTR with DSA which were tested with SABs, the median (IQR) DSA MFI was 16000 (2500-22 000).

On univariate analysis RTR exhibiting DSA had a significantly lower eGFR compared with RTR without DSA (median [IQR] eGFR, 33 [29-55] vs 48 [38-62] mL/min per 1.73 m^2^; *P* = 0.01). DSA seropositivity was not directly associated with any significant alteration in peripheral blood B cell numbers or subset proportions on univariate analysis (data not shown).

There was no difference in the expression of any of the RISET or ITN tolerance signature markers between those who were DSA seropositive and seronegative except *TCL1A*, expressed at a lower level in those who were positive for DSA (data not shown).

A logistic regression method was employed to test for an association between immunosuppression and the presence of DSA. A model was constructed to include previously identified risk factors for DSA development. As DSA have been described in the context of chronic graft decline,^21-23^ renal function (as measured by eGFR) was also included as a covariate.

Azathioprine use was associated with the presence of DSA independent of previously identified risk factors (Table 6). There was also an association between declining eGFR and the presence of DSA, independent of other covariates.

**TABLE 6:**
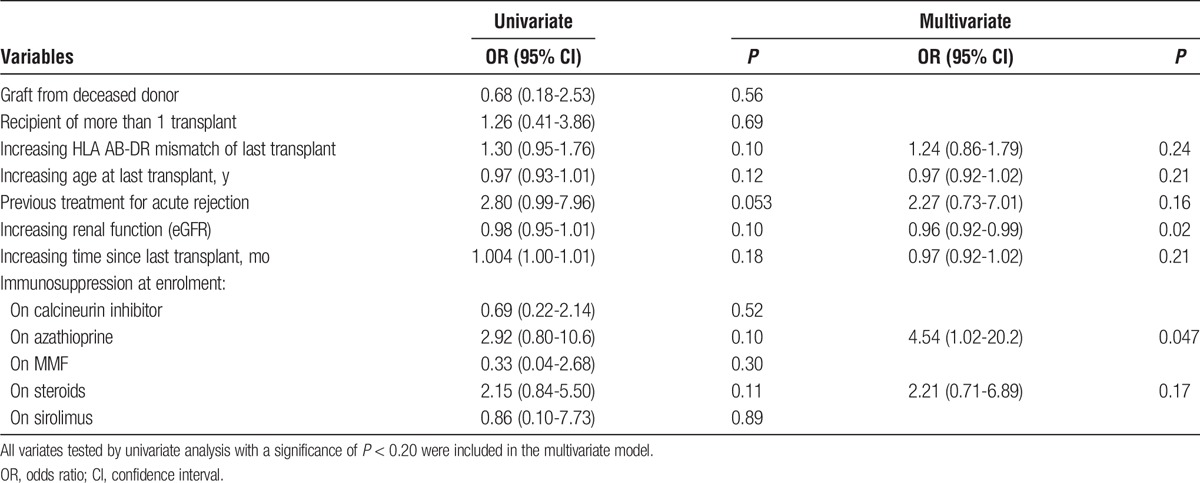
Multivariate analysis of factors associated with the presence of DSA

These results suggested a discrepancy between flow cytometry and gene expression data, indicating increased humoral responses in those treated with calcineurin inhibitors, and the lack of association with IgG DSA. We hypothesized that RTR taking calcineurin inhibitors generate primary IgM responses but fail to generate long lasting IgG memory responses, which require T cell help and germinal center formation.^24,25^ To test this hypothesis quantification of total serum IgG and IgM was assessed using the same model as previously, using each individual immunosuppressant, age at enrolment and time since last transplant as covariates.

**FIGURE 4:**
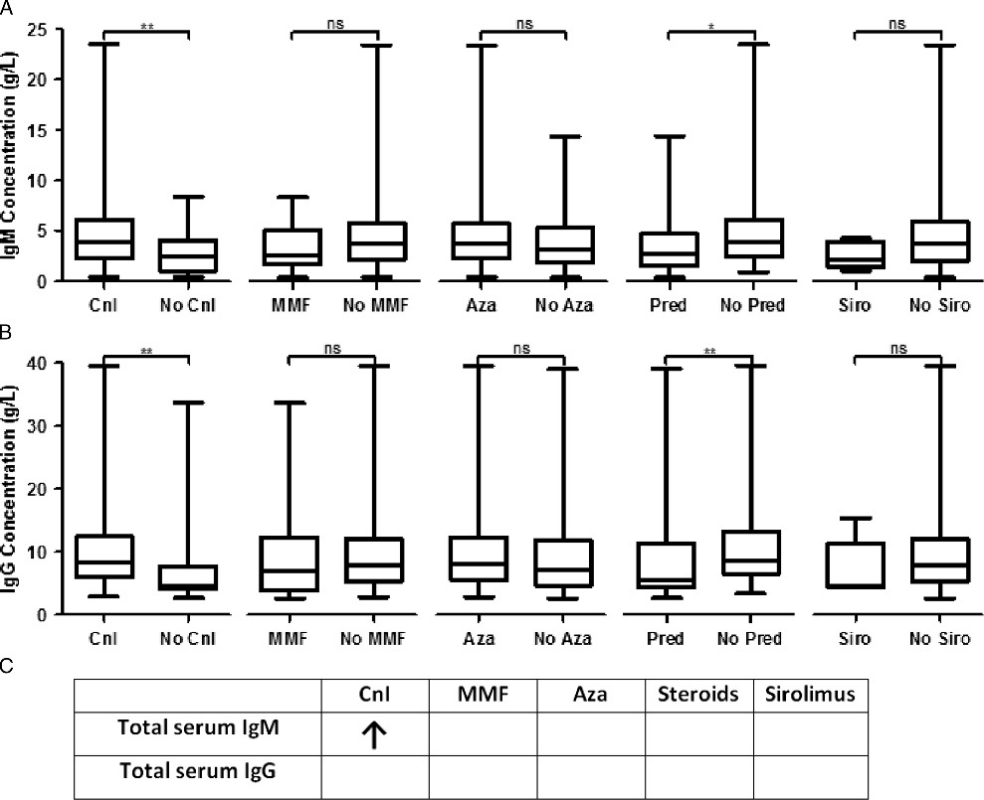
Calcineurin inhibition is associated with increased total serum IgM but not IgG concentration on multivariate analysis. A, Univariate analysis of serum IgM concentration stratified by immunosuppression. B, Univariate analysis of serum IgG concentration stratified by immunosuppression. C, Table of multivariate analysis of effect of immunosuppression on immunoglobulin levels. Results were generated as for Table 4, using log-transformed total antibody concentrations. Significance was taken as *P* < 0.05. Actual values are given in **Table S6, SDC,** http://links.lww.com/TXD/A32. “ns” not significant, *P < 0.05, **P < 0.01.

On univariate analysis, calcineurin inhibition and prednisolone therapy were associated with opposing alterations in the concentration of both serum IgM and IgG. However, when adjusted for other covariates only calcineurin inhibition was independently associated with an increase in total serum IgM concentration. This was not associated with an increase in total serum IgG (Figure 4).

Taken together, these results suggest that although both calcineurin inhibition and azathioprine use are associated with phenotypic changes in the circulating B-cell compartment, only azathioprine is independently associated with the development of humoral responses against the allograft. Calcineurin inhibition was associated with an increase in serum IgM levels, suggesting that the increased proportion of plasmablasts seen on immunophenotyping may be short-lived, early response plasmablasts, the formation of which is not inhibited by calcineurin inhibition. However, this may not translate to long-lived responses and the formation of high-affinity IgG DSA as the T cell help required for sustained humoral immunity and class switch is lacking.

## DISCUSSION

SOpT can be only diagnosed retrospectively upon immunosuppression cessation at present. The ability to identify the development of SOpT prospectively would enable targeted immunosuppression minimization which could reduce therapy-associated complications, such as calcineurin toxicity, cardiovascular disease, and malignancy.^5^ SOpT has been predominantly identified in RTR who are several years posttransplant and so we focused on a long-term cohort where this phenomenon might be maximal.

The association between azathioprine and a reduction in circulating B cell numbers in humans is a relatively novel finding. A study analysing RTRs for the presence of the ITN tolerance signature looked at 8 RTR with long-term transplants and found that azathioprine use was associated with reduced numbers of total and naïve B cells.^17^ We previously found that azathioprine was associated with a reduction in the number of circulating transitional and naive, but not memory, B cells on univariate analysis of RTR.^16^ Administration of azathioprine to mice also results in a reduction in B-cell populations.^26^ In a study of patients with the autoimmune condition systemic lupus erythematosus azathioprine but not MMF was found to be associated with a reduction in circulating naive and transitional B cell numbers on univariate analysis.^27^ We expand upon these observations by demonstrating that the effect of azathioprine on immature B-cell populations is independent of the potentially confounding effect of other immunosuppressive agents, age and duration of immunosuppression.

The original comparator population for the development of the ITN signature was a population of RTR taking calcineurin inhibitors or sirolimus.^9^ *IGKV1D-13*, *IGLL1*, and *IGKV4-1* were expressed at comparable levels in healthy controls and tolerant RTR, but were suppressed in RTR receiving “standard immunosuppression” (consisting of triple therapy of calcineurin or mTOR inhibition, an antiproliferative, and prednisolone). As previously reported elsewhere, we found the amplification of *IGLL1* to be unreliable.^17^ Previous work has suggested that relative expression of the remaining 2 gene signatures, *IGKV1D-13* and *IGKV4-1*, is sufficient to distinguish tolerant and nontolerant RTR in a small cohort.^17^ In this same article, it was also noted that RTR taking calcineurin inhibitors for a significant period were generally more likely to develop a phenotype in keeping with SOpT but that this phenomenon was not seen in azathioprine treated long-term RTR. We confirm this finding in a larger long-term cohort.

Newell et al^9^ found the ITN tolerance signature associated with an increase in transitional and naïve B cells and hypothesized that the upregulation of these genes in operational tolerance may be because of peripheral editing of the BCR receptor. Both *IGKV4-1* and *IGKV1D-13* code for proteins that contribute to immunoglobulin structure.^28,29^ *IGKV1* and *IGKV4* are gene families within the variable region of κ light chains: upregulation of expression of these would be expected to be seen in response to activation-induced maturation of B cells and antibody secretion.^28,30^ Both *IGKV1D-13* and *IGKV4-1* expression correlated significantly with the absolute number and percentage of plasmablasts in peripheral blood (**Table S7, SDC,** http://links.lww.com/TXD/A32). Thus, increased expression of these 2 genes may reflect the increasing presence of plasmablasts in circulating blood. Recently a population of CD19^+^CD138^hi^ ‘regulatory plasma cells’ have been described in mice, acting through secretion of IL-10 and IL-35.^31^ These have not yet been described in humans. It is presumed these would be located in germinal centers outside the circulation and so would not be directly quantified by the ITN tolerance signature.

It was azathioprine rather than calcineurin inhibition that was associated with the presence of donor-specific anti-HLA IgG antibodies after adjustment for previously reported risk factors. The loss of immature B cell populations, a subset of which may have regulatory function,^32^ may favor the development of long lasting, class-switched, alloresponses within the germinal center. In contrast, cyclosporin was associated with upregulation of genes relating to immunoglobulin production but not associated with DSA formation. This may be because calcineurin inhibitors mediate their effects indirectly upon B cells. Cyclosporin has been demonstrated in vitro not necessarily to inhibit B cells directly but may have an inhibitory effect on the T cell help necessary for long-term, high-affinity antibody formation and through reduction of the production of cytokines necessary for germinal center formation and B-cell differentiation, such as IL-2 and IL-21.^24,25,33-36^ The findings here are the first to provide *in vivo* support for this.

Our results highlight the poor correlation between antibody levels and short-lived circulating plasmablast and memory B-cell numbers, as the main source of IgG DSA is from long-lived antibody-secreting plasma cells in the bone marrow.^30,37,38^ The apparent effect of calcineurin inhibition upon mature, effector B-cell populations is a novel and significant finding requiring further mechanistic study.

Our data suggest that sirolimus may also have an effect on aspects of both tolerance signatures. This interpretation should be viewed with caution because only a small subset of RTR were taking this agent; given that this reached significance despite the small number of participants analyzed, this may suggest a major effect.

Certain immunosuppressants may favor the development of SOpT, whereas others will not. Azathioprine may “redirect” the immune system away from operational tolerance, with markers of SOpT appropriately absent in those taking azathioprine due to the increased predisposition towards a humoral response. However, that azathioprine should appropriately affect the RISET but not the ITN signature is concerning. A poor correlation between signatures has been recently recognised elsewhere. A recent meta-analysis of all previous studies that have elucidated genetic signatures of SOpT highlighted that few genes were commonly distributed across the studies, and these few did not possess discriminative value for identifying tolerance.^39^

Our findings suggest the need to identify novel signatures of operational tolerance which are unaffected by the immunosuppressive regimen before these are used in the clinical setting to guide treatment decisions posttransplant. Haynes et al^40^ tested a method of quantifying the indirect pathway of allorecognition in a “spectrum” of RTR, ranging from syngeneic grafts between monozygotic twins to chronic rejectors, but including stable recipients on immunosuppression and a cohort of operationally tolerant RTR. The response to alloantigen correlated with predicted alloreactivity but correlated poorly with the probability of tolerance using the RISET signature. This suggests that this signature may not accurately reflect other mechanisms that may be relevant in the setting of SOpT. Rebollo-Mesa et al^41^ have recently published data in support of our own in an independent cohort, demonstrating the effect of azathioprine and corticosteroids on the RISET signature, as well as proposing a novel signature that may be unaffected by immunosuppression. It remains to be seen whether this new signature correlates to clinical outcomes in validation studies.

Whether the depletion of B cells in those taking azathioprine is reversible on cessation is unclear, though experience with NK cells suggests it is.^42^ A mechanistic explanation for why these specific genes in both signatures were significantly altered in the setting of operational tolerance has been lacking. The data presented here may provide an explanation for this.

The study presented here does have certain limitations. For the purpose of analysis, “calcineurin inhibitors” were taken as a single group. Cyclosporin and tacrolimus both inhibit calcineurin but via different mechanisms.^43^ In both cases, this leads to a failure to express IL-2 after TCR stimulation. However, unlike cyclosporin, tacrolimus may also act via other pathways and therefore may be more potent in inhibiting IL-2 production.^44^

This study was a cross-sectional analysis and as such one must be cautious about assuming causality. We have been careful to describe associations rather than direct causation; longitudinal and mechanistic studies are needed to confirm a link between azathioprine use and DSA formation. This study also suffers from a degree of survival bias, as long-term RTR have, by definition, avoided development of severe alloreactivity or other mechanisms that may lead to chronic allograft failure.

It is unclear whether our findings can be extrapolated backward to earlier in the posttransplant time course. However, a number of studies have suggested that the effect of immunosuppression on lymphocyte populations, and gene expression is relatively rapid in onset and corrects upon immunosuppression cessation.^19,41,42,45^ We attempted to account for any potential cumulative effect of immunosuppression by adjusting for total duration of immunosuppression in our multivariate analyses. Notably, Moreso et al^17^ reported an increase in *IGKV1D-13* expression across the groups taking a calcineurin inhibitor for 1, 5, and 10 years, though this was limited to univariate analysis. The ability to discriminate operational tolerance from nontolerant early posttransplant would be desirable and most clinically relevant because it is this group who would benefit most from immunosuppression reduction or cessation, before the development of immunosuppression-related complications.

The cohort in this study is generally immunosuppressed using agents that are less commonly used in current clinical practice and few received induction therapy. However, this also represents the group where SOpT has been most commonly described but also the group where the most morbidity due to chronic immunosuppression occurs and therefore where markers to guide immunosuppression reduction are most needed.

Finally, the pathogenicity of the DSA detected in this study is unknown. The prevalence of DSA in this study is not dissimilar to that described in studies of cohorts much earlier posttransplant and using other methods of DSA detection.^21,23,46-48^ Although these DSA are associated with poorer graft function, an association with increased rate of graft loss will only become clear with longitudinal study.

In conclusion, we demonstrate that azathioprine may be independently associated with a reduction in circulating naive and transitional B-cell populations, but does not appear to alter mature B-cell populations. In contrast to other immunosuppressive agents, azathioprine may be associated with the development of graft-directed antibodies, and it is possible that this relates to dysregulation of the B-cell compartment and alteration of germinal center populations. In contrast, we interpret our data to indicate that calcineurin inhibition is associated with an increase in the number of postgerminal center B cells and plasmablasts. However, our data suggest the possibility that calcineurin inhibition prevents class switching and the development of long-lasting IgG responses, though may not impact upon IgM responses. Immunosuppression may have an effect on the previously described biomarkers of SOpT and suggests limited clinical applicability in their current form.

## Supplementary Material

SUPPLEMENTARY MATERIAL
