# Supplementary material for: Application of Operational Tolerance Signatures Are Limited by Variability and Type of Immunosuppression in Renal Transplant Recipients: A Cross-Sectional Study
Source: Transplant Direct. 2016 Dec 21;3(1):e125. doi: 10.1097/TXD.0000000000000638 (PMC5361564; doi:10.1097/TXD.0000000000000638)
Supplement: SUPPLEMENTARY MATERIAL [file txd-3-e125-s001.docx]

# Supplementary information

**Full methods**

The conduct of the study was approved by an NHS research ethical committee prior to commencement (reference 12/WS/0288) and was conducted according to the principles of the Declaration of Helsinki. Written consent was provided prior to enrolment. The study is reported according to STROBE guidelines.

**Patient recruitment and clinical data collection**

Long-term renal transplant recipients with stable graft function and without recent non-cutaneous malignancy were recruited at routine clinical follow-up. Inclusion and exclusion criteria are detailed below. Clinical data were collected using medical and transplant records and pathology results. eGFR was calculated using the four-variable ‘Modified Diet in Renal Disease’ equation [[46](#_ENREF_46)].

| Inclusion Criteria | Exclusion Criteria |
| --- | --- |
| Male and female RTR aged greater than 18 years old. | Unable to provide informed consent to participate |
| A stable, functioning renal transplant (defined as serum creatinine increased <30% above baseline value in preceding 12 weeks). | Previous invasive malignancy during the last five years (except cutaneous basal cell carcinoma)* |
| Have provided informed consent to participate | Evidence of systemic infection at time of recruitment (see below) |
|  | Transplant recipient of any organ other than kidney previously, or recipient of a syngeneic renal allograft |
|  | Previous evidence of human immunodeficiency virus (HIV) infection |
|  | Less than one year post-transplant |

***Table S1:*** *Inclusion and exclusion criteria*

**Peripheral blood mononuclear cell (PBMC) extraction and lymphocyte phenotyping**

PBMC were extracted from chilled blood within 4 hours of venepuncture. Briefly, PBMCs were isolated by density-gradient centrifugation and stained using a cocktail of antibodies (Supplementary Table S1). Data were acquired using a Navios flow cytometer and analyzed using Kaluza version 1.4 (both Beckman Coulter, Wycombe, UK) and FlowJoX (TreeStar, Inc). The total lymphocyte count from simultaneous routine haematology laboratory testing was used to calculate absolute cell counts.

**RNA isolation and gene expression analysis**

Total RNA was extracted from thawed whole blood stored in RNA stabilisation solution (‘Tempus’ tubes, Life Technologies, Paisley, UK) at -80^O^C using a magnetic bead (‘MagMAX’, Life Technologies) method according to manufacturer’s instructions. During this process crude RNA is treated with both a protease and a DNAse. Isolated RNA purity was assessed by spectrophotometry (Nanodrop 2000, Thermo Fisher Scientific, Loughborough, UK); all samples yielded RNA with a 260/280nm optical density ratio of greater than 2.00. RNA was stored at -80^O^C prior to reverse transcription.

Complementary DNA (cDNA) was generated using recombinant moloney murine leukemia virus reverse transcriptase and random hexamers (Life Technologies) using a starting quantity of 1μg of total RNA. All gene expression assays had been previously tested for efficient and linear amplification using serial dilution.

Quantitative/Real-time PCR (qPCR) was undertaken on 30ng cDNA in duplicate using either inventoried assays or using custom primers and probes (Supplementary Table S2). Relative gene expression was normalised to beta-glucuronidase (*GUSB)*, using the 2^-ΔCq^ method (*HPRT*, *18S* and *GAPDH* were also assessed as candidate reference genes). No template controls were run in parallel. qPCR conditions were 50^O^C for 2 minutes, then 95^O^C for 10 minutes, then 45 cycles of 95^O^C for 15 seconds and 60^O^C for 1 minute.

**Anti-HLA antibodies detection and definition**

Anti-HLA antibodies were detected using solid-phase Luminex bead assays (One Lambda Inc., California, US), according to the validated protocols used for clinical samples by the Transplant Immunology and Immunogenetics Laboratory, Churchill Hospital, Oxford. 10μl thawed serum was incubated for 30 minutes with 5μl beads conjugated to HLA antigens. After washing, beads were incubated with Phycoerythrin (PE)-conjugated goat anti-human IgG for 30 minutes. Samples were analysed using a Luminex 100 IS fluorescence detector system (Luminex Corp., Texas, US).

All samples were first assessed using LabScreen Mixed Screen (LSM12) beads. Samples with a positive result were confirmed using Class I (LS1PRA) or Class II PRA (LS2PRA) beads. If HLA specificities cannot be resolved definitively, further testing was performed using Class I (LS1A04) or Class II (LS2A01) single antigen beads (SAB). Samples with high negative control fluorescence were pre-treated with AdsorbOut beads (One Lambda Inc.) to reduce non-specific binding. All samples requiring single antigen bead testing were pre-treated with 0.3% ethylenediaminetetraacetic acid (EDTA).

Samples were run with positive and negative control samples and control beads. The mean fluorescence intensity (MFI) on each individual sample bead was normalised to a negative control bead within each sample and a negative control sample provided by the manufacturer. For SAB, the cut-off for positivity was an MFI of 1500, representing the clinical threshold used at the Oxford Transplant Centre.

Antibodies to HLA-A, -B, ,-C, -DR and -DQ were assessed as this information was available for the majority of participants. For 3 donor-recipient pairs the donor HLA Class II alleles were unknown, so only their HLA Class I data were assessed.

**Serum immunoglobulin concentration quantification**

Serum immunologlobulin concentration was determined by ELISA (Total Human IgM and IgG Ready- SET-Go kits, eBioscience). Thawed sera were diluted (1:10,000 for IgM and 1:500,000 for IgG) and incubated in duplicate for two hours in 96-well flat bottom plates (Nunc MaxiSorp, Thermo Scientific) pre-coated with capture antibody. Plates were washed and incubated for one hour at room temperature with horseradish peroxidase-conjugated detection antibody, before further washing and addition of 3,3′,5,5′-tetramethylbenzidine substrate solution for 15 minutes. The substrate reaction was stopped with 2N sulphuric acid and the plate read immediately at a wavelength of 450nm using a microplate reader (eMax system, Molecular Devices, Wokingham, UK).

**Statistical analysis**

Analyses were performed on Graphpad Prism for Windows 5.03 (Graphpad, San Diego, USA) or SPSS 20 (IBM Corp., New York, USA). Continuous variables are reported as median (interquartile range) unless specified otherwise. Hazard ratios are reported as hazard ratio (95% confidence interval). Categorical variables are reported as number (percentage of group).

Inter-group comparison was performed using the non-parametric two-tailed Mann-Whitney or Kruskal-Wallis tests. For categorical variables the chi-squared test or Fisher’s exact test were used. Where the Kruskal-Wallis test was significant, a subsequent post-hoc Dunn test was applied.

The ‘forced entry’ method of regression was used for all analyses. Linear regression for interaction of immunosuppression with the signatures of tolerance was performed using normally-transformed variables where appropriate. All variables were transformed using log-transformation. Odds ratios were calculated by logistic regression.

Throughout the study a p-value of less than 0.05 was considered significant, unless indicated otherwise. In order to prevent Type II (false positive) errors due to multiple testing, where appropriate a Bonferroni correction was applied; the adjusted threshold for significance is indicated where used.

| **Specificity-conjugate** | **Clone** | **Supplier** |
| --- | --- | --- |
| CD3-eFluor 450 | UCHT1 | eBioscience, Hatfield, UK |
| CD38-APC | HIT2 |  |
| CD20-APC-eFluor 780 | 2H7 |  |
| CD4-ECD | SFCI12T4D11 | Beckman Coulter, High Wycombe, UK |
| CD19-Krome Orange | J3-119 |  |
| CD8-PerCP-Cy5.5 | SK1 | BD Biosciences, Oxford, UK |
| CD27-FITC | M-T271 |  |
| CD127-PE | HIL-7R-M21 |  |
| CD25-PE-Cy7 | MA251 |  |
| CD69-Alexa Fluor 700 | FN50 |  |
| CD24-PE | ML5 |  |
| IgM-PE-Cy5.5 | G20-127 |  |
| IgD-PE-Cy7 | IA6-2 |  |
| FoxP3-Alexa Fluor 647 | 259D | Biolegend, London, UK |

***Table S2:*** *Antibodies used for flow cytometry analysis.*

| **Assay Target** | **Sequence or assay ID** | **Supplier** |
| --- | --- | --- |
| *FOXP3* | 5’- AAG TGG CCC GGA TGT GAG A -3’ (FWD)  5’- CAT TGT GCC CTG CCC TTC T -3’ (REV)  6FAM- ACT TCC TCA AGC ACT GCC AGG CGG –TAMRA (Probe) | Sigma (probe)  Eurogentec (primers) |
| *AMANN* | 5’- CGG CCA GAA GTT ATG GAG ACT TAC -3’ (FWD)  5’- CTT AGG CCT GAA TAG CCT CCA TTC -3’ (REV)  6FAM- TGG GAA GCC GTA GAG GCC TTG GAA A –TAMRA (Probe) | Sigma (probe)  Eurogentec (primers) |
| *TLR5* | Hs00152845_m1 | Life Technologies |
| *PNOC* | Hs00173823_m1 | Life Technologies |
| *SH2D1B* | Hs01592483_m1 | Life Technologies |
| *SLC8A1* | Hs00253432_m1 | Life Technologies |
| *HS3ST1* | Hs01099196_m1 | Life Technologies |
| *FCRL1* | Hs00364705_m1 | Life Technologies |
| *FCRL2* | Hs00229156_m1 | Life Technologies |
| *MS4A1* | Hs00544818_m1 | Life Technologies |
| *TCL1A* | Hs00172040_m1 | Life Technologies |
| *CD79B* | Hs00236881_m1 | Life Technologies |
| *HPRT* | 5’- AGT CTG GCT TAT ATC CAA CAC TTC G -3’ (FWD)  5’- GAC TTT GCT TTC CTT GGT CAG G -3’ (REV)  6FAM- TTT CAC CAG CAA GCT TGC GAC CTT GA –TAMRA (Probe) | Sigma (probe)  Eurogentec (primers) |
| *GUSB* | Hs99999908_m1 | Life Technologies |
| *18S* | 5' GCC CGA AGC GTT TAC TTT GA – 3’ (FWD)  5' TCC ATT ATT CCT AGC TGC GGT ATC – 3’ (REV)  6FAM- AAA GCA GGC CCG AGC CGC C –TAMRA (Probe) | Life Technologies |
| *GAPDH* | NM_002046.3 | Life Technologies |
| *IGLL1* | Hs00252263_m1 | Life Technologies |
| *IGKV4-1* | AIN1E62 | Life Technologies |
| *IGKV1D-13* | 5’ GGG CTT CTG CTG CTC TGG 3’ (FWD)  5’ TGG AGA CTG GGT CAA CTG GAT 3’ (REV)  6FAM- CCA GGT GCC AGA TGT G –NFQ (Probe) | Life Technologies |

***Table S3: Primers and probes used for gene expression analysis.***

|  | CnI | MMF | Aza | Steroids | Sirolimus |
| --- | --- | --- | --- | --- | --- |
| CD19/CD3 ratio | 0.18 (-0.06 – 0.43, 0.13) | 0.03 (-0.25 – 0.33, 0.79) | -0.31 (-0.47 – 0.08, 0.007) | 0.03 (-0.13 – 0.18, 0.79) | -0.12 (-0.57 – 0.13, 0.22) |
|  |  |  |  |  |  |
| %CD4^+^ CD25^int^ | 0.21 (-0.04 – 0.33, 0.12) | 0.09 (-0.15 – 0.29, 0.52) | 0.12 (-0.08 – 0.22, 0.37) | 0.06 (-0.09 – 0.15, 0.61) | 0.15 (-0.09 – 0.44, 0.19) |
| %CD4^+^ CD25^+^(effector) | 0.37 (0.07 – 0.39, 0.006) | 0.13 (-0.10 – 0.29, 0.32) | 0.13 (-0.07 – 0.20, 0.31) | 0.10 (-0.06 – 0.15, 0.36) | 0.18 (-0.04 – 0.44, 0.10) |
|  |  |  |  |  |  |
| *FOXP3*/*AMANN* ratio | -0.02 (-0.21 – 0.19, 0.91) | -0.09 (-0.32 – 0.15, 0.47) | -0.14 (-0.25 – 0.07, 0.27) | -0.06 (-0.16 – 0.09, 0.60) | -0.03 (-0.33 – 0.24, 0.77) |
|  |  |  |  |  |  |
| *TCL1A* expression | 0.06 (-0.22 – 0.38, 0.60) | -0.06 (-0.44 – 0.27, 0.63) | **-0.41 (-0.70 – -0.22, <0.001)** | -0.22 (-0.42 – -0.04, 0.02) | -0.23 (-0.95 – -0.09, 0.02) |
| *TLR5* expression | -0.07 (-0.17 – 0.09, 0.56) | 0.15 (-0.05 – 0.26, 0.18) | 0.10 (-0.06 – 0.15, 0.37) | **0.44 (0.11 – 0.27, <0.001)** | 0.10 (-0.09 – 0.29, 0.30) |
| *SH2D1B* expression | -0.11 (-0.29 – 0.09, 0.29) | -0.09 (-0.33 – 0.13, 0.38) | **-0.70 (-0.72 – -0.41, <0.001)** | 0.01 (-0.11 – 0.13, 0.89) | 0.04 (-0.21 – 0.35, 0.60) |
| *HS3ST1* expression | -0.08 (-0.24 – 0.13, 0.54) | 0.04 (-0.19 – 0.25, 0.78) | -0.09 (-0.20 – 0.09, 0.46) | **-0.36 (-0.32 – -0.09, 0.001)** | -0.12 (-0.41 – 0.12, 0.27) |
| *MS4A1* expression | 0.05 (-0.17 – 0.25, 0.69) | -0.01 (-0.26 – 0.23, 0.91) | -0.30 (-0.39 – -0.06, 0.01) | -0.16 (-0.24 – 0.03, 0.12) | -0.28 (-0.72 – -0.12, 0.007) |
| *SLC8A1* expression | -0.13 (-0.14 – 0.05, 0.32) | -0.04 (-0.13 – 0.09, 0.73) | 0.03 (-0.06 – 0.08, 0.81) | 0.28 (0.02 – 0.14, 0.006) | 0.11 (-0.06 – 0.20, 0.31) |
| *FCRL1* expression | 0.19 (-0.04 – 0.35, 0.12) | 0.08 (-0.16 – 0.31, 0.53) | -0.28 (-0.35 – -0.04, 0.02) | -0.006 (-0.13 – 0.12, 0.95) | -0.02 (-0.31 – 0.26, 0.87) |
| *CD79B* expression | 0.06 (-0.10 – 0.17, 0.64) | -0.04 (-0.19 – 0.13, 0.72) | -0.29 (-0.25 – -0.03, 0.01) | -0.18 (-0.17 – 0.01, 0.07) | -0.18 (-0.37 – 0.02, 0.08) |
| *PNOC* expression | -0.07 (-0.20 – 0.12, 0.61) | -0.06 (-0.23 – 0.14, 0.63) | -0.07 (-0.16 – 0.09, 0.60) | -0.09 (-0.14 – 0.06, 0.41) | -0.25 (-0.49 – -0.04, 0.02) |
| *FCRL2* expression | 0.16 (-0.09 – 0.40, 0.22) | 0.03 (-0.26 – 0.33, 0.84) | -0.16 (-0.33 – 0.07, 0.21) | -0.05 (-0.20 – 0.12, 0.63) | -0.06 (-0.46 – 0.26, 0.58) |

Table S4: The effect of individual immunosuppression upon the RISET tolerance signature. *Results are generated using linear regression on log-transformed variables. Significance was adjusted for multiple testing using Bonferonni correction and taken to be <0.004. ‘CnI’, calcineurin inhibitor; ‘MMF’, mycophenolate mofetil; ‘Aza’, azathioprine. Statistically significant variables are shown in bold.*

|  | CnI | MMF | Aza | Steroids | Sirolimus |
| --- | --- | --- | --- | --- | --- |
| *IGKV4-1* expression | **0.34 (0.15 – 0.68, 0.002)** | **-0.22 (-0.64 – -0.01, 0.04)** | -0.02 (-0.23 – 0.20, 0.87) | -0.07 (-0.24 – 0.11, 0.45) | **-0.24 (-0.88 – -0.11, 0.01)** |
| *IGKV1D-13* expression | **0.40 (-0.21 – 0.74, 0.001)** | -0.20 (-0.60 – 0.03, 0.07) | -0.03 (-0.24 – 0.19, 0.80) | -0.02 (-0.19 – 0.15, 0.85) | **-0.20 (-0.80 – -0.03, 0.03)** |

Table S5: The effect of immunosuppression upon the ITN tolerance signature. *Results were generated as for Table S4. Significance was taken as p<0.05. Statistically significant variables are shown in bold.*

|  | CnI | MMF | Aza | Steroids | Sirolimus |
| --- | --- | --- | --- | --- | --- |
| Total serum IgM | **0.31 (0.06 – 0.52, 0.01)** | -0.01 (-0.29 – 0.26, 0.92) | 0.02 (-0.17 – 0.20, 0.87) | -0.16 (-0.27 – 0.03, 0.13) | 0.06 (-0.25 – 0.43, 0.60) |
| Total serum IgG | 0.13 (-0.08 – 0.26, 0.30) | -0.08 (-0.27 – 0.14, 0.54) | -0.01 (-0.14 – 0.14, 0.54) | -0.17 (-0.20 – 0.02, 0.10) | -0.04 (-0.30 – 0.20, 0.72) |

Table S6: The effect of immunosuppression upon total serum antibody levels***.*** *Results were generated using log-transformed total antibody concentrations. Significance was taken as p<0.05. Statistically significant variables are highlighted in bold.*

| **Correlations** | | | |  |
| --- | --- | --- | --- | --- |
|  | | Number of plasmablasts | Percentage of plasmablasts | |
| Expression of *IGKV4-1* | Pearson Correlation | 0.397 | 0.273 | |
|  | Sig. (2-tailed) | <0.001 | 0.003 | |
| Expression of *IGKV1D-13* | Pearson Correlation | 0.431 | 0.427 | |
|  | Sig. (2-tailed) | <0.001 | <0.001 | |

***Table S7: correlation between number and percentage of plasmablasts in circulating blood and ITN tolerance signature variables.***


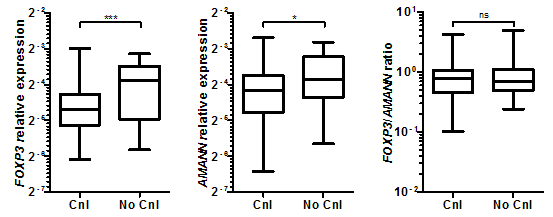


***Figure S1: Calcineurin inhibition reduces both* FOXP3 *and* AMANN *relative expression, leading to no alteration in* FOXP3*/*AMANN *ratio.*** *Expression is relative to* GUSB*. ‘ns’, not significant, *p<0.05, ***p<0.001. ‘CnI’, calcineurin inhibitor.*
